# Autistic traits and individual brain differences: functional network efficiency reflects attentional and social impairments, structural nodal efficiencies index systemising and theory-of-mind skills

**DOI:** 10.1186/s13229-020-00377-8

**Published:** 2021-01-21

**Authors:** Subhadip Paul, Aditi Arora, Rashi Midha, Dinh Vu, Prasun K. Roy, Matthew K. Belmonte

**Affiliations:** 1 MIND Research Network, 1101 Yale Blvd NE, Albuquerque, NM 87106 USA; 2 National Brain Research Centre, NH-8, Nainwal Mode, Manesar, 122051 India; 3 Centre for Cognitive Neuroscience, Universität Salzburg, Kapitelgasse 4-6, 5020 Salzburg, Austria; 4 National Institute of Mental Health and Neuro Sciences, Hosur Road, Bangalore, 560029 India; 5 Department of Psychology, University of Oslo, Harald Schjelderups hus, Forskningsveien 3A, 0373 Oslo, Norway; 6 Department of Psychology, Chaucer Bldg., Nottingham Trent University, Shakespeare Street, Nottingham, NG1 4FQ UK; 7 School of Biomedical Engineering, Indian Institute of Technology (Banaras Hindu University), Varanasi, 221005 India; 8 The Com DEALL Trust, 224, 6th ‘A’ Main Road, near Specialist Hospital, 2nd Block, HRBR Layout, Bangalore, 560043 India

**Keywords:** Autism, Dimensional, Social, Attention, Theory-of-mind, fMRI, DTI, Functional connectivity, Graph theory

## Abstract

**Background:**

Autism is characterised not only by impaired social cognitive ‘empathising’ but also by superior rule-based ‘systemising’. These cognitive domains intertwine within the categorical diagnosis of autism, yet behavioural genetics suggest largely independent heritability, and separable brain mechanisms. We sought to determine whether quantitative behavioural measures of autistic traits are dimensionally associated with structural and functional brain network integrity, and whether brain bases of autistic traits vary independently across individuals.

**Methods:**

Thirty right-handed neurotypical adults (12 females) were administered psychometric (Social Responsiveness Scale, Autism Spectrum Quotient and Systemising Quotient) and behavioural (Attention Network Test and theory-of-mind reaction time) measures of autistic traits, and structurally (diffusion tensor imaging) and functionally (500 s of 2 Hz eyes-closed resting fMRI) derived graph-theoretic measures of efficiency of information integration were computed throughout the brain and within subregions.

**Results:**

Social impairment was positively associated with functional efficiency (*r* = .47, *p* = .006), globally and within temporo-parietal and prefrontal cortices. Delayed orienting of attention likewise was associated with greater functional efficiency (*r* = − .46, *p* = .0133). Systemising was positively associated with global structural efficiency (*r* = .38, *p* = 0.018), driven specifically by temporal pole; theory-of-mind reaction time was related to structural efficiency (*r* = − .40, *p* = 0.0153) within right supramarginal gyrus.

**Limitations:**

Interpretation of these relationships is complicated by the many senses of the term ‘connectivity’, including functional, structural and computational; by the approximation inherent in group functional anatomical parcellations when confronted with individual variation in functional anatomy; and by the validity, sensitivity and specificity of the several survey and experimental behavioural measures applied as correlates of brain structure and function.

**Conclusions:**

Functional connectivities highlight distributed networks associated with domain-general properties such as attentional orienting and social cognition broadly, associating more impaired behaviour with more efficient brain networks that may reflect heightened feedforward information flow subserving autistic strengths and deficits alike. Structural connectivity results highlight specific anatomical nodes of convergence, reflecting cognitive and neuroanatomical independence of systemising and theory-of-mind. In addition, this work shows that individual differences in theory-of-mind related to brain structure can be measured behaviourally, and offers neuroanatomical evidence to pin down the slippery construct of ‘systemising’ as the capacity to construct invariant contextual associations.

**Supplementary Information:**

The online version contains supplementary material available at 10.1186/s13229-020-00377-8.

## Background

The twenty-first century science and public rhetoric of autism have been dominated by a shift from exclusively categorical construal as a disease condition to a recognition of dimensional autistic traits throughout the general population. In this regard, autism and autistic traits have mirrored axes of variation within other complex neuropsychiatric conditions such as schizophrenia and schizotypy [1], or obsessive-compulsive disorder and obsessionality, compulsivity and ordering [2]. Although specific patterns of variation may converge on general graph-theoretic hub territories such as prefrontal cortex in schizophrenia, the devil is in the details, and studies do not in general agree [3, 4] on how exactly these dimensional trait measures map onto dimensional variations in brain connectivity. Categorical autism is distinguished by brain dysconnectivity [5, 6], and as the construal of autism has extended to dimensional traits, so have such dimensional behavioural and cognitive traits begun to be related to dimensional variation in brain connectivity [7]. These dimensional relationships complement and interact with categorical differences [8], no doubt presenting developmental endpoints of complex interactive specialisation [9, 10]. But are broadly defined and broadly surveyed social and perceptual ‘autistic’ traits fundamentally neurally related to autism itself, or are they but reflections of individual variation? [11].

Neurophysiological variables have been shown to covary with behavioural measures of such traits, but these behavioural measures have been dominated by ones that focus on social communication in particular, most commonly the well validated and oft used Social Responsiveness Scale [12], and by informant-report or even self-report surveys rather than experimental measures. Although the canonical social communicative result on autism is apparent impairment in theory-of-mind, the past three and a half decades have not yielded convergence on any single scale by which this deficit ought to be measured. In general, different tests of theory-of-mind seem to agree less with each other and more in terms of their various confounds [13], such as working memory and language. The main approach to resolving the binary outcome of the original, ‘Sally-Anne’ test of theory-of-mind into a continuous measure has been to sum binary item scores across multiple test scenarios, with checks for comprehension [14, 15]; a complementary approach is to average reaction times across repeated trials of ToM versus non-ToM scenarios [16]. As the former, accuracy-based method tends to yield ceiling effects in non-autistic populations; in this study of dimensional autistic traits, we implement the latter, reaction-time approach, within a motivating, game-based context [17] whose graphical presentation minimises verbal confounds.

Social communication can be the most obvious of autistic traits but is far from the only axis of variation, and experimental measures can yield information more direct and domain-specific, complementary to that provided by surveys. Dimensional brain-behaviour relationships may vary across behavioural domains and across types of behavioural assay, and questions remain as to whether and how such relationships differ across sensory/perceptual, attentional, social cognitive, and verbal tasks, and across survey and experimental behavioural measures. Might behaviourally measured correlations between social and non-social autistic traits [18, 19] persist into the realm of brain function? Or do these dimensional traits have independent brain bases reflecting their mostly independent heritability [7, 20–22]? And do survey and experimental behavioural measures yield similar results?

At the same time as behavioural measures can be extended, so can metrics of brain structure and function. The field has witnessed a shift from raw measures of diffusion anisotropy and temporal correlation to derived graph-theoretic metrics that address more specifically the networks subserving neural information transfer [23]. Pathways between brain regions can be represented as a graph-theoretic complex networked system, where regions are vertices and pathways are edges of the network [24]. Such mathematical representation of white-matter pathways is known as a ‘structural connectome’ [24, 25], defined by anatomical connections. The functional connectome, on the other hand, represents relations between brain regions’ functional activities [26], where graph edges are defined as cross-correlations between time series of functional activations. Graph-theoretic complex network measures allow characterisation of structural and functional brain networks at global and nodal/regional levels [27].

Whole-brain associations between the graph-theoretic resting-state functional network measures, DTI-derived measures of localised white-matter integrity, and psychometrics including the Social Responsiveness Scale and Autistic Spectrum Screening Questionnaire have been explored previously [28], linking autistic traits with reduced average local (but not global) functional efficiency (a graph-theoretic measure of how well neural networks integrate information from disparate sources) overall, and in right posterior cingulum in particular. Associations between the Social Responsiveness Scale scores and rs-fMRI connectivity were also explored in a region-of-interest approach centred on rostral anterior cingulum [29], finding reduced correlation with mid-insula and heightened correlation with lateral occipital cortex, superior parietal cortex and angular gyrus. However, structural connectome measures as a function of dimensional autistic traits have only recently begun to be assayed [30].

This preliminary study applies *both survey and experimental* measures to identify dimensional variation in brain-behaviour relationships, across *both social and non-social* behavioural and cognitive domains of autistic traits in the normal population, using graph-theoretic metrics derived from *both functional (resting fMRI) and structural (DTI)* measures of brain connectivity. We ask which combinations of behavioural (survey and experimental, social and non-social) and brain (structural and functional) measures might be sensitive to such brain-behaviour dimensions. The straightforward hypotheses are an association of broad measures of autistic traits with reduced local functional network integrity (low efficiency, low clustering, long path length) across the entire brain [28], alongside similar effects within anatomical regions functionally associated with specific constructs and their measures (e.g. impaired theory-of-mind with right temporoparietal junction [31], slowed orienting of attention with intraparietal sulcus [32], impaired executive control with anterior cingulum [28, 33], superior systemisimg with posterior parietal cortex and impaired empathising with frontotemporal cortices [34]).

## Methods

### Subjects

Thirty right-handed volunteers (mean age ± standard deviation: 27.29 years ± 2.88, 18 males, 12 females) participated in the study for small monetary reimbursement. All participants had normal or corrected-to-normal vision, and no history of psychological or neurological disorders. Written informed consent was obtained from participants.

### Behavioural measures

Five questionnaires and three computer-based psychometric tests along with two tests of verbal fluency were administered (Table 1, Additional file 1). All participants completed the adult version of the Autism Spectrum Quotient (AQ [35]). On the basis of our previous work [18] in which granular scoring of the AQ made a more sensitive correlate of other measures of autistic traits, the AQ was scored on a symmetric 4-point Likert scale. AQ subcategory scores were recorded, both the social subscore AQSoc comprising attention switching (AQAttSw), communication (AQComm), imagination (AQImag) and social skills (AQSS), and the details/patterns subscore (AQDet). Participants’ self-reported ability to understand others’ intentions, predict their behaviour, and respond with appropriate emotions were measured with the Empathy Quotient (EQ) [36].

**Table 1:** Mean and standard deviation of all behavioural measures

Behavioural measures	Female	Male	Total
AQ			
Mean	− 18.92	− 19.56	− 19.24
*SD*	*27.16*	*19.18*	*23.17*
EQ			
Mean	45.75	39.39	42.57
*SD*	*12.73*	*11.20*	*11.97*
SQ			
Mean	64.92	74.56	69.74
*SD*	*15.89*	*17.25*	*16.57*
SSQ			
Mean	5.75	3.50	4.63
*SD*	*2.45*	*1.50*	*1.98*
SRS			
Mean	33.00	42.76	37.88
*SD*	*12.88*	*25.67*	*19.27*
RMET			
Mean	27.64	25.44	26.54
*SD*	*4.95*	*4.68*	*4.81*
COWA			
Mean	49.00	43.11	46.06
*SD*	*14.21*	*7.94*	*11.08*
AnNT			
Mean	18.00	16.50	17.25
*SD*	*5.59*	*4.59*	*5.09*
FC-EFT			
Mean	11410.41 (ms)	8815.5 (ms)	10112.95 (ms)
*SD*	*5561.96 (ms)*	*5002.31 (ms)*	*5282.13 (ms)*
ANT (alerting effect)			
Mean	48.25 (ms)	51.70 (ms)	49.97 (ms)
*SD*	*34.53 (ms)*	*28.56 (ms)*	*31.54 (ms)*
ANT (orienting effect)			
Mean	58.166 (ms)	48.05 (ms)	53.11 (ms)
*SD*	*37.64 (ms)*	*20.97 (ms)*	*29.31 (ms)*
ANT (conflict effect)			
Mean	116.33 (ms)	114.64 (ms)	115.49 (ms)
*SD*	*38.56 (ms)*	*40.14 (ms)*	*39.35 (ms)*
ANT (grand mean effect)			
Mean	656.33 (ms)	620.52 (ms)	638.43 (ms)
*SD*	*60.32 (ms)*	*34.16 (ms)*	*47.24 (ms)*
Second-order ToM			
Mean	− 5.20 (s)	− 0.063 (s)	− 2.63 (s)
*SD*	*4.95 (s)*	*10.61 (s)*	*7.78 (s)*
Egocentric first-order ToM			
Mean	6.984 (s)	1.183 (s)	4.08 (s)
*SD*	*14.01 (s)*	*18.65 (s)*	*16.33 (s)*
Allocentric first-order ToM			
Mean	3.94 (s)	0.548 (s)	2.24 (s)
*SD*	*5.160 (s)*	*11.477 (s)*	*8.31 (s)*
WASI (verbal IQ)			
Mean	109.42	109.67	109.54
*SD*	*9.89*	*7.90*	*8.90*
WASI (performance IQ)			
Mean	102.00	109.17	105.58
*SD*	*6.66*	*7.08*	*6.87*
WASI (full-scale IQ)			
Mean	106.83	110.89	108.86
*SD*	*7.52*	*7.54*	*7.53*

*AQ* Autism Spectrum Quotient, *EQ* empathy quotient, *SQ* systemizing quotient, *SSQ* Sensory Sensitivity Questionnaire, *SRS* Social Responsiveness Scale, *RMET* Reading the Mind in the Eyes Test, *COWA* Controlled Oral Word Association test, *AnNT* Animal Names Test, *FC-EFT* forced-choice version of the Embedded Figures Test, *ANT* Attention Network Task, *WASI* Wechsler Abbreviated Scale of Intelligence

The Systemizing Quotient (SQ) [37] assessed the drive to build contexts from individual parts and details. ‘Systemizing’, having been a somewhat nebulously defined construct, deserves some explication. Baron-Cohen et al. define a ‘system’, in this context, as “something that takes inputs, which can then be operated on in variable ways, to deliver different outputs in a rule-governed way” [37]. More specifically, a system might map between concrete motor outputs and sensory inputs, as with a fidget spinner, or between mechanical causes and effects, as with an engine, or between logical parameters input and results output, as with a computer, or between books or records and their linearly or hierarchically ordered classification numbers, as in a library. The face validity in relation to autistic preferences and expertise at classifying, ordering and predicting within explicitly defined systems seems clear enough. However, the SQ’s construct validity as a measure of drive to understand rule-based input-output relations has been called into question by its lack of any strong relationship to mathematical skill [38]. The SQ’s questions focus on construction, spatial mappings, component parts, component mechanisms and processes, and taxonomies (described by Ling et al. [39] as ‘DIY’, ‘topography’, ‘structure’, ‘technicity’ and ‘taxonomy’, respectively)—all processes that emphasise spatially and temporally invariant, static and therefore predictable relations of parts and details to frames and contexts, and thus reflect an autistic cognitive style of *bricolage*, in which abstract and general representations are effortfully, extensionally constructed bottom-up from the underlying details and instances [40]. Baron-Cohen’s systemizing construct thus is not so much a drive to understand rule-based input-output relations as it is a skill of constructing *invariant contextual associations*. SQ scores map onto population-level sex differences in cognitive traits related to autism [41], males being on average more prone to the SQ’s systemizing approach to cognition, and females being greater at the cognitive empathy tapped by same group’s ‘Empathizing Quotient’.

Autistic sensory traits were assessed by the Sensory Sensitivity Questionnaire (SSQ) [42–45]. At the time of data collection, few self-report or informant-report measures of sensory processing quick enough to be acceptable to subjects in this multi-measure study were yet available (see Table 3 of DuBois et al. [46]), and those that were available had been used in categorical contrasts of autistic and non-autistic populations (e.g*.* [47]) rather than as dimensional measures—a trend that continues today. Thus, the selection of a sensory measure was based on a combination of free availability and face validity for autism, its use as a dimensional measure being necessarily an extrapolation.

To assess autistic social communicative traits, the Social Responsiveness Scale- Adult (SRS) [12] was completed by subjects’ social partners (e.g. spouse, parent, longtime friend). The SRS was developed as a measure of subthreshold autistic traits and has accumulated an extensive history and norms for use as such. One subject did not return the SRS.

Social cognition also was measured by the ‘Reading the Mind in the Eyes’ Test (RMET) [48]. The RMET originally was developed as a test of the ability to infer another’s mental state, its face validity being established by its covariance with other quantitative measures of autism spectrum conditions and autistic traits. It has been applied widely as a dimensional measure of autistic traits (e.g. [18, 49]). Later work demonstrates that the RMET measures more the ability to name emotions [50], and verbal ability in general [51], although with this interpretive caveat the RMET remains useful as a measure of social cognitive function in the broad sense. The test comprises 36 photographs of the eye region, for each of which participants choose the one of four words that best describes what the person in the picture is feeling or thinking.

Perceptual disembedding was measured by a forced-choice version of the Embedded Figures Test (FC-EFT) [52, 53]. Subjects were asked to locate the embedded shape as rapidly as possible with a 50-s timeout interval, pressing the number key 1 for the shape on the left or the number key 9 for the shape on the right. The score is the mean latency of correct responses. The EFT has been applied in many studies of dimensional autistic traits (e.g. [18]), though a review of these by Cribb et al. [54] suggests that the EFT may be most effective when applied categorically or between extremes rather than along a continuum of dimensional variation.

The Attention Network Test Revised (ANT-R) [33, 55] combines the Posner visual attention task [56] with a visual spatial flanker task [57] to measure alerting (temporal effect of cueing), orienting (spatial effect of cueing), and executive control over conflicts between percept-action mappings (effect of flanker congruence), the final two of which, at least, are demonstrably perturbed in autism [58]. Scores were computed as simple differences of mean reaction times in trials with correct responses: alerting, central cue minus no cue; orienting, spatial cue minus central cue; conflict, no cue with incongruent flankers minus no cue with congruent flankers. The ANT has been validated against self-report measures of individual differences in attentional control [59], though not to our knowledge in terms of autistic traits *per se*.

Phonetic association fluency was measured by the Controlled Oral Word Association (COWA) test [60]. The COWA evaluates the spontaneous, timed production of words beginning with a given letter. In three 1-min trials, participants were asked to generate as many words as possible beginning with ‘F’, ‘A’ and ‘S’, respectively, excluding proper names and names of numbers. The score was the mean tally of qualifying words, excluding repetitions, across the three trials. Subjects also completed the Animal Names Test [61], a semantic fluency test in which subjects are asked to generate in a 1-min timed trial as many animal names as possible, excluding the names of fish, birds and snakes. The score is the total number of qualifying animal names, excluding repetitions. Similar phonetic and semantic fluency tasks have been oft applied in measurement of individual differences [62].

Egocentric and allocentric [63] first-order theory-of-mind (ToM), and second-order ToM, were assessed as reaction time differences between conditions in a graphical version of the Sally-Anne test. This task was implemented as a computer game [17] (see Supplementary Methods) wherein Sally is a friendly spaceship captain, Anne is a space pirate, the ball is a cache of resources for the player’s space station, and the basket and the box each are one of four planets distinguished by spatial position, colours and texture. The resulting no-theft, unobserved-theft and observed-theft vignettes were presented mainly graphically, supplemented by simple textual narrative at the bottom of the display. The subject was reminded that Sally would always steer her spaceship where she *thought* the cache was, and was asked to set a course to meet up with her by moving a trackpad cursor up, down, left or right to one of the four planets.

To assess general intelligence, participants were tested with the Wechsler Abbreviated Scale of Intelligence (WASI) [64]. The WASI’s four subtests estimate verbal comprehension and perceptual reasoning abilities that contribute to general intelligence.

### Image acquisitions

MR image acquisitions were performed using a 3 Tesla Philips Achieva scanner with eight-channel head coil. The head was immobilised using cushions and straps. During the resting-state fMRI data acquisition, participants were asked to keep their eyes closed, relax and not think about anything specific, but to avoid sleeping. All participants confirmed that they did not sleep and did not come close to falling asleep during the scan. Resting-state Blood Oxygen Level Dependent (BOLD) signals were acquired using a 3D PRESTO (principles of echo shifting with a train of observations) sequence [65, 66] with the following parameters: field of view (FOV) 256 mm × 256 mm × 140 mm, voxel dimension 4 mm × 4 mm × 4 mm, 1000 time points, dynamic scan time 500 ms, repetition time (TR) 22 ms, echo time (TE) (shifted) 32 ms, SENSE p reduction = 2, SENSE s reduction = 2, flip angle (FA) 9°. This high-frequency (2 Hz) sampling minimises aliasing of high-frequency (~ 0.1–1 Hz) cardiac and respiratory oscillations into the slower (0.01–0.1 Hz) spontaneous fluctuations of BOLD signal. However, these fMRI parameters yield lower anatomical contrast compared to T1-weighted images. This 9° functional scan therefore was followed by a similar PRESTO scan at 25° flip angle for use in spatially co-registering the PRESTO images against T1-weighted anatomical images [67]. Lastly, a 3D T1-weighted image (Additional files 2, 3, 4, 5 and 6) was acquired using a Turbo Field Echo sequence with the following parameters: FOV 240 mm × 240 mm × 160 mm, voxel dimension = 1.0 mm^3^ isotropic, TR 8 ms, TE 3.69 ms, flip angle 8°.

Diffusion-weighted images were obtained using a spin-echo (SE) echo-planar imaging (EPI) sequence, FOV 224 mm × 224 mm × 140 mm, voxel dimension = 2 mm × 2 mm × 2 mm, TR/TE = 9386/58 ms, diffusion gradient timing (Δ/*δ* = 28.9/17.8 ms, *b* = 1000 s/mm^2^, 32 directions, with fat suppression using Spectral Presaturation by Inversion Recovery (SPIR). The sequence was repeated three times (total gradient directions = 96) in order to improve the quality of the diffusion-weighted signals. Fifteen volumes with no diffusion weighting (*b* = 0 s/mm^2^) were also acquired.

### Preprocessing

Individual fMRI scans were spatially re-aligned to the last functional volume using Statistical Parametric Mapping (SPM) software. Then, the high-contrast 25° PRESTO scan was spatially co-registered to these realigned fMRI scans and each T1-weighted image was linearly transformed onto it using the default setting of FLIRT (FMRIB’s Linear Image Registration Tool). T1-weighted images were segmented into grey matter (GM), white matter (WM) and cerebrospinal fluid (CSF) maps using FAST (FMRIB’s Automated Segmentation Tool), and these maps were transformed to the realigned PRESTO scans using *applyxfm* function of FSL (FMRIB Software Library). Nearest-neighbour interpolation was used so as to avoid introducing partial-volume tissue categories whilst spatially transforming the segmented maps. Further processing took place in DPARSF (Data Processing Assistant for Resting-State fMRI) [68]. The first 10 volumes of the realigned fMRI scans were discarded and the remaining fMRI time series with 990 time points were de-trended. Averaged WM and CSF signals were from the fMRI time series using the realigned tissue-segmented maps as masks. This and head motion parameters were regressed out (Additional file 7) from the fMRI time series. High-frequency respiratory and cardiac oscillations (0.1–1 Hz) and low-frequency scanner drift (< 0.01 Hz) were removed by band-pass filtering at 0.01–0.1 Hz [69]. The fMRI datasets were not spatially smoothed, as smoothing is inherent in spatially averaging the time series from each region of interest (described in the subsection on “Network construction”).

We have concatenated the three sets of diffusion imaging data and the gradient tables. In order to correct the distortion of diffusion-weighted images due to eddy currents and head motion, the diffusion-weighted images of each participant were registered to the respective first *b* = 0 image using affine transformation. Rotational components corresponding to each diffusion-weighted volume were extracted from the transformation matrix, and the B-matrix was rotated using the extracted rotation vector in order to correct for head motion [70].

### Modelling of diffusion-weighted signals

Diffusion-weighted signal in each voxel was modelled considering multiple fibre orientations in the voxel. Metropolis-Hastings Markov Chain Monte Carlo sampling was used for estimation of model parameters and a Bayesian method, Automatic Relevance Determination (ARD), was applied to determine whether the diffusion-weighted signals of a voxel should be represented by a single-fibre or a multiple-fibre model [71]. Diffusion-weighted data were modelled using the *bedpostx* function of FDT (FMRIB’s Diffusion Toolbox) with default values of the parameters.

### Network construction

A network with *N* nodes and *K* edges can be denoted by a graph (*G(N,K)*) [72]. The rows and columns of the *N* × *N* adjacency matrix represent the nodes and each element (*w*_*ij*_) of this matrix denotes the link between the *i*th and the *j*th nodes of the network. We describe the procedures to construct adjacency matrices to represent the functional and structural connectivity networks below.

#### Correlation matrix

Using FNIRT (FMRIB’s non-linear image registration tool), the ICBM152 T1 template was non-linearly registered to each participant’s preprocessed fMRI scans, by using the PRESTO-aligned T1-weighted image as the registration target. This spatial transformation was then applied to the AAL-90 atlas label map using FSL’s *applywarp* function, with nearest-neighbour interpolation to retain the values of the atlas labels. The resulting AAL-90 map registered to each individual participant’s functional scans was masked using that participant’s similarly registered tissue segmentation map to select only grey matter voxels.

Using this grey matter AAL-90 label map, the averaged time series corresponding to each AAL-90 brain area was extracted from the preprocessed fMRI scans. The symmetric 90 × 90 correlation matrix (Additional file 8) for each participant was constructed by calculating the zero-lagged Pearson correlation coefficient between all pairs of BOLD time series. Each element (*w*_*ij*_) of the correlation matrix is the correlation between the time series extracted from the *i*th and the *j*th AAL-90 regions. Zeroes have been assigned to the diagonal.

#### Connection matrix

Similarly to the preprocessing of the functional images described above, T1-weighted images were rigidly registered to the corresponding first *b* = 0 image using FLIRT, segmented GM maps were linearly transformed to the corresponding *b* = 0 space, the *b*=0-registered T1-weighted images were non-linearly registered to the ICBM152 T1 template using FNIRT and AAL-90 label maps were warped to the *b* = 0 images using the inverted non-linear transformations with nearest-neighbour interpolation. The voxels of the label map that corresponded to grey matter in the b=0-registered segmented GM map were selected for tractography.

Probabilistic tractography was executed from each brain area to the other 89 brain areas using FDT (FMRIB’s Diffusion Toolbox). For every sampled streamline fibre at the seed voxel, a sample direction was selected from the local direction distribution. Moving 0.5 mm to a new location along the sample direction, a new sample direction was selected from the direction distribution at that new location. Five thousand streamline fibres were sampled from each seed voxel in the probabilistic tractography framework. Let us consider a brain area comprising *n* voxels. Dividing the number of fibres passing through that area by *n* × 5000 yields the connection probability from the seed area to the given area. However, the connection probability of the *i*th brain area to the *j*th brain area is not necessarily equal to the connection probability of the *j*th brain area to the *i*th brain area. We have calculated undirected connection probability between those two areas (*P*_*ij*_) by taking the average of those two probabilities. A symmetric connection matrix (Additional file 8) of dimension 90 × 90 for each participant was constructed by performing probabilistic tractography from all 90 brain areas. Each element (*w*_*ij*_) of the connection matrix denotes the undirected connection probability between the *i*th and the *j*th AAL-90 regions. Zeroes have been assigned to the diagonal.

### Network metrics

Using the brain connectivity toolbox [27], we have calculated the functional and the structural network metrics for each participant from the corresponding correlation matrix and the connection matrix respectively, the coefficient in each matrix cell serving as an adjacency weight for the corresponding pair of AAL-90 regions. As we are interested not in whole-group patterns but in individual differences, weights have not been thresholded [6]; any non-zero weight represents some measure of adjacency. This strategy avoids the potential of artefactually generating disconnected graph components which could skew the measures in some subjects. In selecting these metrics, we have taken a cue from previous studies of autism and autistic traits: Billeci et al. [30] used characteristic path length and clustering coefficient, and Jakab et al. [28] used local and global efficiency. A deficit in “small-world” network topology, defined as a combination of high density of connections for computation within local neural neighbourhoods and direct connection routes for information transfer between these neighbourhoods, has been cited as a distinguishing characteristic of the autistic brain [73]. Small-world topology is maintained by minimising ‘wiring’ connections whilst maintaining strong clustering; small characteristic path length and large clustering coefficient, therefore, constitute perhaps the most straightforward graph-theoretic metrics of complexity. Global and local efficiencies are related to path length and clustering, respectively, but efficiency can be a more powerful derived metric for empirical data, because its summation of reciprocals of path lengths gives weight to hubs and parallel connections rather than to disconnected regions and serial connections [74].

#### Strength

Strength of the *i*th node (${s}_i^w$) of the network is denoted by the summation over the edge weights (*w*_*ij*_) of all links to the *i*th node:

1 $$ {s}_i^w={\sum}_{j\in N}{w}_{ij} $$

The value of the nodal strength reveals the significance of that node in the network [27].

#### Characteristic path length

The characteristic path length of a network is defined as the average shortest path length between all pairs of nodes in the network [27]:

2 $$ {L}^w=\frac{1}{N}{\sum}_{\mathrm{i}\in N}\frac{\sum_{i\in N,j\ne i}{d}_{ij}^w}{N-1} $$

where ${d}_{ij}^w$ (the inverse of connection strength, *w*_*ij*_) denotes the element of weighted distance matrix (*d*^*w*^). Characteristic path length is a global measure of integration in a network.

#### Clustering coefficient

The weighted clustering coefficient of the *i*th node of the network (${C}_i^w$) is defined [75] as

3 $$ {C}_i^w=\frac{\sum_{j,m\in N}{\left({w}_{ij}{w}_{im}{w}_{jm}\right)}^{\frac{1}{3}}}{k_i\left({k}_i-1\right)} $$

where *k*_*i*_ stands for the weighted degree, *k*_*i*_ = ∑_*j* ∈ *N*_*w*_*ij*_. When *k*_*i*_ = 0 or 1, a zero value is assigned to the clustering coefficient of that node. The clustering coefficient is a network-based measure of segregation which denotes the ability of specialised processing within densely connected brain regions [27, 76]. The nodal clustering coefficient represents how strongly a node is clustered with its neighbouring nodes. The global clustering coefficient of a network is calculated by taking the average of the clustering coefficients over all the nodes of the network:

4 $$ {C}^w=\frac{1}{N}{\sum}_{i\in N}{C}_i^w $$

The clustering coefficient of a network (*C*^*w*^) characterises the level of interconnectivity of the network.

#### Global and local efficiencies

Global and local efficiencies measure the integration of information from distributed brain areas and estimate how well these brain areas communicate. Global efficiency of the network is computed using the following expression [77]:

5 $$ {E}_{\mathrm{glob}}^w=\frac{1}{N}{\sum}_{i\in N}\frac{\sum_{j\in N,j\ne i}{\left({d}_{ij}^w\right)}^{-1}}{N-1} $$

On the other hand, local efficiency of the *i*th node of the network is the global efficiency of the neighbourhood of that node [27]:

6 $$ {E}_{\mathrm{loc},i}^w=\frac{1}{2}{\sum}_{i\in N}\frac{\sum_{j,m\in N,j\ne i}{\left({w}_{ij}{w}_{jm}{\left[{d}_{jm}^w\left({N}_i\right)\right]}^{-1}\right)}^{\frac{1}{3}}}{k_i\left({k}_i-1\right)} $$

where ${d}_{jm}^w\left({N}_i\right)$ denotes the shortest path between the *j*th and *m*th nodes which are within the neighbourhood of the *i*th node.

### Statistical analyses

Behavioural measures and the functional and structural network metrics were z-transformed. Behavioural measures were correlated with the network metrics using multi-linear regression. Age, gender, full-scale IQ, head-motion effect (average frame-wise displacement [78]) and brain volumes were considered as confounding factors, and were removed from the neuroimaging-based measures using multiple linear regression. Spearman correlation coefficients between the resulting residuals of the neuroimaging-based measures and the behavioural measures were computed across all subjects. Tail probabilities, one-sided with respect to whichever tail was nearest, were estimated using permutation testing (as in the supplementary information of [79]) as [min(|{ρ_rand_ | ρ_obs_>ρ_rand_}|, |{ρ_rand_ | ρ_obs_<ρ_rand_}|)+1]/N where ρ_obs_ is the Spearman correlation computed from the actual data and the ρ_rand_ are the *N* = 10000 Spearman correlations computed on the *N* = 10000 random permutations of the data (Additional file 9). Statistical significances of the correlations between the nodal/regional neuroimaging-based measures and each behavioural measure were thresholded at 5% false discovery rate (FDR) [80], except in the case of theory-of-mind measures with an a priori hypothetical association with brain regions in the right temporoparietal junction. Effect sizes are indicated by absolute values of the correlation coefficients.

## Results

**Fig. 1:**
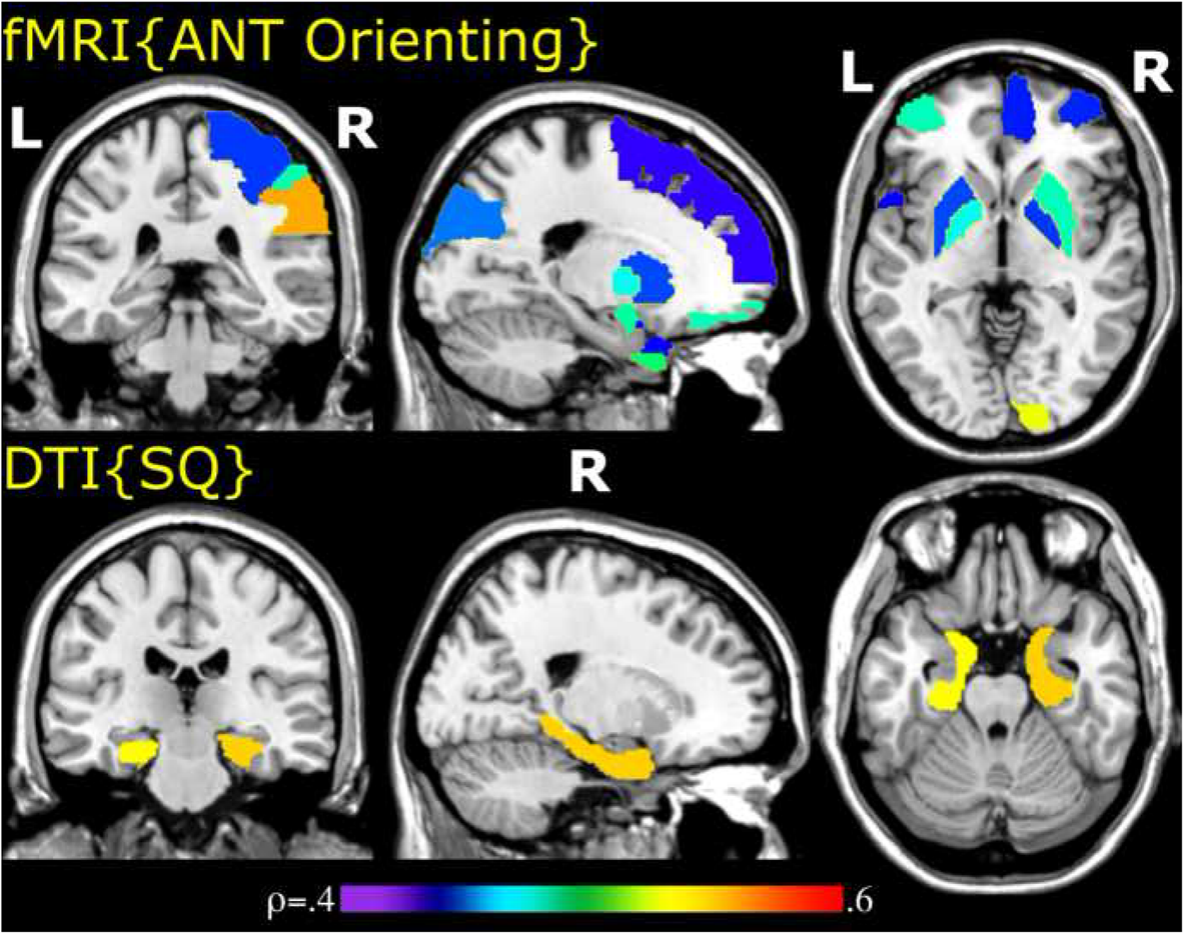
Examples of functional and structural, experimental and survey results. Regional correlations between nodal functional network efficiencies and Attention Network Test orienting scores (top), and between nodal structural network clustering coefficients and Systemizing Quotient scores (bottom). Sizes of the regions reflect of AAL-90 parcellations and are not individually any indication of network extents. Orienting is associated with a functional attention network comprising basal ganglia and frontal, parietal and visual cortices, overlapping substantially with that for social responsiveness (not shown), whereas systemising is associated with structural contextual-association networks within parahippocampal cortices. Right supramarginal gyrus, the cortical region most strongly associated with attention orienting functionally, also is the region associated with theory-of-mind structurally

Global functional clustering, characteristic path length and efficiency correlated with Social Responsiveness Scale (Table 2). This global association was driven by nodal strength, clustering and efficiency in a network spanning most of the cerebral cortex (as well as putamen and globus pallidus), with strength differences in prefrontal, medial-anterior temporal and temporoparietal cortices (full details in Table 3). Global functional clustering, characteristic path length and efficiency also correlated with the orienting score on the Attention Network Test (Table 2). This global association was driven by nodal clustering (but not significantly by nodal strength or efficiency) in a similarly widespread cerebral network encompassing a smaller number of regions in prefrontal, temporal and parietal cortices as well as putamen and globus pallidus (full details in Table 4, illustrated in Fig. 1). We have not observed any significant associations between global (*p* > 0.05) or nodal (pFDR > 0.05) functional network measures and other behavioural measures (Table 2).

**Table 2:** Spearman correlation coefficients between questionnaire/behavioural measures and global functional network measures

Behavioural measure	Clustering coefficient ρ (*p*)	Characteristic path length ρ (*p*)	Efficiency ρ (*p*)	Behavioural measure	Clustering coefficient ρ (*p*)	Characteristic path length ρ (*p*)	Efficiency ρ (*p*)
2 ^o^ ToM	− 0.121 (0.257)	0.087 (0.321)	− 0.202 (0.142)	Reading the Mind in the Eyes	0.170 (0.185)	− 0.220 (0.119)	0.233 (0.108)
Egocentric 1^o^ ToM	− 0.030 (0.445)	− 0.015 (0.464)	− 0.003 (0.496)	Controlled Oral Word Association	− 0.032 (0.436)	− 0.024 (0.448)	0.033 (0.434)
Allocentric 1^o^ ToM	− 0.036 (0.430)	0.017 (0.468)	− 0.006 (0.492)	Animal names	− 0.123 (0.251)	0.094 (0.300)	− 0.042 (0.405)
AQ Soc	0.308 (0.053)	− 0.277 (0.069)	0.277 (0.071)	Embedded figures	− 0.029 (0.438)	0.055 (0.382)	0.039 (0.419)
AQ Det	− 0.106 (0.277)	0.127 (0.237)	− 0.077 (0.329)	ANT alerting	− 0.202 (0.142)	0.100 (0.304)	− 0.206 (0.136)
Empathy quotient	− 0.175 (0.173)	0.187 (0.159)	− 0.145 (0.218)	**ANT orienting**	− 0.423 (0.010)*	0.441 (0.007)*	− 0.406 (0.013)*
Systemizing quotient	− 0.006 (0.488)	0.021 (0.459)	0.011 (0.475)	ANT conflict	− 0.095 (0.313)	0.170 (0.186)	− 0.115 (0.275)
Sensory Sensitivity Questionnaire	0.049 (0.410)	0.013 (0.461)	0.008 (0.491)	ANT grand mean	− 0.002 (0.488)	0.028 (0.427)	− 0.017 (0.458)
**Social Responsiveness Scale**	0.455 (0.0075)*	− 0.387 (0.018)*	0.467 (0.006)*				

*p < 0.05

**Table 3:** Spearman correlation coefficients between Social Responsiveness Scale scores and nodal functional network measures

Region	Nodal strength ρ (***p***)	Nodal clustering ρ (***p***)	Nodal efficiency ρ (***p***)
L precentral gyrus		0.442 (0.008)	0.452 (0.006)
R precentral gyrus		0.468 (0.005)	0.514 (0.002)
L superior frontal gyrus		0.549 (0.001)	0.523 (0.001)
L superior frontal (orbital)	0.545 (0.001)	0.423 (0.011)	0.434 (0.010)
R superior frontal (orbital)	0.558 (0.0006)	0.466 (0.004)	0.449 (0.006)
R middle frontal gyrus		0.510 (0.003)	0.510 (0.002)
L middle frontal (orbital)	0.603 (0.0004)	0.636 (0.0002)	0.639 (0.0002)
R middle frontal (orbital)	0.547 (0.001)	0.518 (0.002)	0.532 (0.001)
L inferior frontal (opercular)		0.382 (0.020)	0.413 (0.013)
R inferior frontal (triangular)		0.446 (0.007)	0.401 (0.015)
L inferior frontal (orbital)		0.440 (0.007)	0.434 (0.009)
R inferior frontal (orbital)		0.389 (0.017)	
L Rolandic operculum		0.397 (0.015)	
R Rolandic operculum		0.490 (0.003)	0.452 (0.006)
L supplementary motor area	0.590 (0.0006)	0.366 (0.023)	0.449 (0.007)
L medial superior frontal gyrus	0.510 (0.003)		
L olfactory		0.396 (0.017)	0.437 (0.008)
R olfactory		0.355 (0.027)	0.365 (0.025)
R superior frontal gyrus (medial)		0.413 (0.013)	0.358 (0.027)
L superior frontal (medial orbital)			0.362 ( 0.024)
R superior frontal (medial orbital)			0.379 (0.021)
L insula		0.409 (0.013)	0.383 (0.019)
R insula		0.377 (0.020)	0.351 (0.029)
L anterior cingulate, paracingulate			0.368 (0.022)
R anterior cingulate, paracingulate		0.437 ( 0.009)	0.376 ( 0.021)
L median cingulate, paracingulate		0.560 (0.0008)	0.468 (0.004)
L posterior cingulate		0.368 (0.024)	0.386 (0.020)
R posterior cingulate		0.372 (0.022)	
R hippocampus	0.576 (0.001)	0.410 (0.011)	0.345 (0.030)
L parahippocampal gyrus		0.405 (0.012)	0.337 (0.030)
R parahippocampal gyrus		0.431 (0.008)	0.412 (0.011)
L amygdala		0.449 (0.006)	0.415 ( 0.013)
R amygdala	0.479 (0.003)	0.418 (0.010)	0.393 (0.016)
L calcarine cortex		0.483 (0.003)	0.461 (0.005)
R calcarine cortex		0.445 (0.007)	0.385 (0.017)
L cuneus		0.388 (0.016)	0.401 (0.014)
R cuneus		0.409 (0.012)	0.381 (0.020)
L lingual gyrus		0.487 (0.003)	0.445 (0.006)
L superior occipital gyrus		0.464 (0.005)	0.492 (0.002)
L fusiform gyrus		0.356 (0.026)	0.363 (0.022)
R fusiform gyrus		0.413 (0.012)	0.408 (0.012)
L postcentral gyrus			0.355 (0.029)
R postcentral gyrus			0.355 (0.027)
L inferior parietal lobule		0.492 (0.003)	0.433 (0.010)
R inferior parietal lobule		0.356 (0.028)	0.393 (0.017)
L supramarginal gyrus	0.478 (0.004)	0.403 (0.014)	0.417 (0.011)
R supramarginal gyrus		0.479 (0.004)	0.443 (0.007)
L angular gyrus	0.572 (0.0006)	0.457 ( 0.006)	0.511 (0.003)
R angular gyrus		0.448 (0.007)	0.445 (0.007)
L precuneus		0.617 (0.0002)	0.567 ( 0.001)
R precuneus		0.374 (0.023)	0.359 (0.026)
L paracentral lobule			0.374 (0.021)
R paracentral lobule		0.419 (0.011)	
L putamen		0.369 (0.023)	
R putamen		0.587 (0.0004)	0.550 (0.0008)
L pallidum		0.486 (0.004)	0.444 (0.008)
R pallidum	0.649 (0.0001)	0.366 (0.021)	
R lenticular nucleus, pallidum			0.357 (0.026)
L thalamus			0.358 (0.026)
L Heschl’s gyrus		0.363 (0.022)	0.354 (0.027)
L superior temporal pole		0.499 (0.003)	0.444 (0.007)
R superior temporal pole		0.354 (0.029)	0.365 (0.023)
L middle temporal gyrus	0.503 (0.003)	0.459 (0.007)	0.473 (0.005)
L middle temporal pole		0.451 (0.007)	0.399 (0.015)
R middle temporal pole		0.466 (0.006)	0.392 (0.016)
R inferior temporal	0.578 (0.0007)	0.417 (0.010)	0.417 (0.011)

**Table 4:** Spearman correlation coefficients between Attention Network Test orienting scores and nodal functional clustering coefficients

Regions	Clustering coefficient ρ (*p*)
R precentral gyrus	− 0.511 (0.003)
L superior frontal	− 0.417 (0.010)
L middle frontal (orbital)	− 0.480 (0.004)
R middle frontal (orbital)	− 0.431 (0.009)
L inferior opercular frontal	− 0.412 (0.012)
R olfactory	− 0.450 (0.006)
R middle orbitofrontal	− 0.429 (0.007)
R gyrus rectus	− 0.439 (0.008)
L amygdala	− 0.485 (0.003)
R amygdala	− 0.479 (0.004)
R calcarine cortex	− 0.553 (0.0008)
L superior occipital gyrus	− 0.445 (0.006)
R postcentral gyrus	− 0.434 (0.007)
R inferior parietal lobule	− 0.481 (0.004)
R supramarginal gyrus	− 0.571 (0.001)
L putamen	− 0.437 (0.006)
R putamen	− 0.480 (0.003)
L pallidum	− 0.471 (0.004)
R pallidum	− 0.434 (0.010)
L superior temporal pole	− 0.424 (0.009)
L middle temporal pole	− 0.494 (0.003)
R middle temporal pole	− 0.455 (0.005)

All results are significant at 5% FDR level

Global structural clustering and efficiency correlated with Systemizing Quotient (SQ) scores (*p* < 0.05) (Table 5). This global association was driven by nodal strength, clustering and efficiency in a network comprising the medial temporal lobes and right temporal pole (full details in Table 6, illustrated in Fig. 1). At the nodal level, allocentric first-order theory-of-mind reaction time difference was associated with efficiency (ρ = − 0.397, one-tailed *p* = 0.0153) and clustering (ρ = − 0.400, one-tailed *p* = 0.014, both uncorrected given a priori association of theory-of-mind with right temporoparietal junction) in right supramarginal gyrus, which was the only brain region to manifest any significant uncorrected correlation with theory-of-mind. We have not observed any significant associations between global (*p* > 0.1) or nodal (pFDR > 0.05) structural network measures and other behavioural measures (Table 5). Global structural characteristic path lengths were not significantly associated with any behavioural measures (*p* > 0.05).

**Table 5:** Spearman correlation coefficients between questionnaire/behavioural measures and global structural network measures

Behavioural measures	Clustering coefficient ρ (*p*)	Characteristic path length ρ (*p*)	Efficiency ρ (*p*)	Behavioural measures	Clustering coefficient ρ (*p*)	Characteristic path length ρ (*p*)	Efficiency ρ (*p*)
2 ^o^ ToM	0.127 (0.248)	− 0.051 (0.394)	0.144 (0.218)	Reading the Mind in the Eyes	− 0.162 (0.195)	0.210 (0.135)	− 0.141 (0.229)
*Allocentric 1*^*o*^ *ToM*	− *0.266 (0.076)*	0.216 (0.130)	− *0.280 (0.065)*	Controlled Oral Word Association	− 0.079 (0.333)	0.114 (0.273)	− 0.073 (0.349)
Egocentric 1^o^ ToM	− 0.205 (0.137)	0.149 (0.216)	− 0.169 (0.181)	Animal names	0.077 (0.342)	− 0.005 (0.487)	0.154 (0.207)
AQ Soc	− 0.104 (0.295)	0.136 (0.235)	− 0.115 (0.273)	Embedded figures	− 0.088 (0.314)	− 0.002 (0.498)	− 0.076 (0.335)
AQ Det	− 0.067 (0.366)	0.107 (0.288)	− 0.063 (0.372)	ANT alerting	0.003 (0.495)	0.056 (0.379)	0.043 (0.415)
Empathy quotient	0.221 (0.124)	− 0.235 (0.106)	0.213 (0.131)	ANT orienting	0.152 (0.210)	− 0.164 (0.196)	0.152 (0.206)
**Systemizing quotient**	0.368 (0.023)*	− 0.256 (0.090)	0.385 (0.018)*	ANT conflict	− 0.052 (0.396)	− 0.041(0.410)	− 0.068 (0.365)
Sensory Sensitivity Questionnaire	0.156 (0.202)	− 0.242 (0.097)	0.107 (0.286)	ANT grand mean	0.293 (0.211)	− 0.283 (0.196)	0.301 (0.207)
Social Responsiveness Scale	0.207 (0.128)	− 0.175 (0.168)	0.219 (0.116)				

*p < 0.05

**Table 6:** Spearman correlation coefficients between Systemizing Quotient (SQ) scores and nodal structural network measures

Regions	Strength vs SQ ρ (*p*)	Regions	Clustering coefficient vs SQ ρ (*p*)	Regions	Efficiency vs SQ ρ (*p*)
L Parahippocampal	0.585 (0.0003)	R Parahippocampal	0.556 (0.0007)	L Parahippocampal	0.556 (0.001)
R Parahippocampal	0.592 (0.0004)	R Middle temporal pole	0.517 (0.0013)	R Parahippocampal	0.564 (0.0005)
R Olfactory	0.555 (0.0007)				

All results are significant at 5% FDR level

## Discussion

We applied both survey and experimental measures, and both functional and structural brain imaging, to assay brain-behaviour relationships in both social and non-social autistic trait dimensions. Various social and non-social measures correlated with both global functional and regional structural network efficiency, although the direction of these correlations was contrary to hypothesis: greater autistic traits tended to associate with greater efficiency. Where correlations were detected, more specific capacities such as systemising and theory-of-mind were related to structure of specific brain regions whereas general or integrative traits such as social responsiveness and attention orienting associated with function of anatomically distributed networks. This work incidentally shows that individual differences in theory-of-mind can be measured using reaction time differences from even small numbers of trials, and introduces a new perspective on the ‘systemising’ construct as the capacity to construct invariant contextual associations.

One non-social survey measure—the Systemizing Quotient—covaried with global structural network efficiency driven especially by medial temporal lobes, and one social experimental measure—theory-of-mind—covaried with nodal efficiency and clustering near right temporoparietal junction. One social cognitive survey measure—the SRS—and one non-social experimental measure—ANT orienting—covaried with global functional network efficiencies. AQSS and AQDet despite tapping a social communicative construct related to the SRS and an attentional construct related on face to the ANT, respectively, bore no significant relationship, and neither did sensory, perceptual, empathic or verbal measures. This pattern of results might have as much to do with the relatively well established validity of the SRS and the ANT as it might with any primacy of social responsiveness and attentional orienting, although the developmental relationship between these two constructs [81, 82] does not escape our notice.

The nodal functional results are less certain than the corresponding global results, because of two sources of variation that render fMRI-based network localisations inherently broad. First, functional connectivity studied across the entire brain emphasises widespread networks and not localised neighbourhoods, because the time series correlations on which it is based are essentially transitive, making the resulting connectivity graph a transitive closure: if, for example, supramarginal gyrus is functionally connected with orbitofrontal cortex, and orbitofrontal cortex is functionally connected with medial temporal lobe, then supramarginal gyrus will to some degree be functionally connected to medial temporal lobe. Second, functional maps at the population level are accurate only in broad strokes; individual differences in functional anatomical boundaries [83] imply that multiple functional subregions are collapsed into single anatomical parcels at the resolution of AAL-90, and thus that multiple functional relationships amongst these regions are likewise collapsed into single edges within any network graph. So it makes sense that the fMRI connectivity results highlight distributed networks associated with domain-general properties such as attentional orienting and a broad measure of social responsiveness.

Both in the case of attentional orienting and in the case of social cognition, these fMRI-based measures found brain networks to be physiologically *more* efficiently organised for individuals higher in autistic traits, even though high autistic traits mean that one is behaviourally *less* efficient at each of these cognitive skills, taking longer to orient attention and being less socially responsive. This disjunction between physiological and behavioural measures of efficiency can be interpreted in at least three ways:

1. Despite the deficits in these two specific behavioural measures, autistic traits can make people more efficient at other aspects of cognition. This scenario is easy to imagine and is enunciated in Asperger’s absent-minded “Professor” whose “besonderen Leistungen” (“unusual achievements”) come hand in hand with “Hilflosigkeit dem praktischen Leben gegenüber” (“helplessness in the face of practical life”) [84], a trope repeated time and again in postmodern literature and media (e.g. [85], p. 6). In terms of neural and cognitive mechanisms, a perturbed excitatory/inhibitory balance [86] produces abnormally low network entropy [87] which when it arises during activity-dependent development evokes abnormal desegregation between networks [88, 89] consistent with the observation of enhanced ‘rich club’ connectivity of network hubs in autism [6, 90]. The cognitive result can be temporally inefficient orienting [91] and spatially inefficient filtering [92]. This ‘sticky’ style of attention may lead to rumination on particular stimuli and details, and to compensatory processing [93] that yields ultimately a more complete style of representation based on *bricolage* [40]; likewise, time and cognitive effort not spent on exhausting and often futile attempts at social understanding may instead be invested in understanding the more tractable world of deterministic systems and rules.
2. fMRI-based connectivity may measure a crude combination of connectivities within anatomically superimposed functional networks that differ in fMRI-indistinguishable parameters such as operating frequency band and cellular physiology. Previous studies of eyes-closed resting EEG taken together show, if nothing else, that the picture of autistic brain connectivity becomes complicated when frequency band is taken into account. Findings include elevated short-range coherence in the theta band and reduced long-range coherence in the lower-alpha band [94], elevated short-range and reduced long-range coherence in delta band correlating with score on the Autism Diagnostic Observation Schedule (ADOS) [73], and reduced delta and theta coherences at all ranges with reduced alpha and beta coherences at some short-range electrode pairs [95]. An eyes-open resting MEG study measuring graph-theoretic relations amongst correlations between signal envelopes in a variety of frequency bands found greater gamma network efficiency but lesser beta efficiency in autism, both categorically and in correlation with ADOS scores, along with greater alpha efficiency categorically [96]; these authors suggested an altered balance between heightened bottom-up, gamma-mediated signalling and attenuated top-down, beta-mediated signalling [97]. Both EEG [98] and intracranial [99] recordings in humans have demonstrated that BOLD fMRI is most positively coupled to gamma oscillations. Viewed through this lens, then, our fMRI connectivity data become consistent with the thesis of heightened bottom-up, gamma-mediated connectivity not only in autism categorically but perhaps also with autistic traits dimensionally. This second case of interpretation is not at all mutually exclusive with the first case above; indeed, such an altered balance between bottom-up and top-down information flow could be the physiological substrate of autistic cognitive superiorities and deficits.
3. There may be a discontinuity between clinically autistic impairment on the one hand and subclinical levels of nominally ‘autistic’ traits that form part of general individual differences on the other. Autistic traits in separate domains of cognitive function tend to be inherited largely independently [100, 101] and constitute distinct domains of function in the non-autistic population [102], but once they cross watershed levels they may begin to synergise, reinforcing each other as development proceeds [19]. The result might be a classic inverted-U dose-response curve: traits that individually and in moderate doses are cognitively adaptive may in combination and in higher doses become cognitively impairing overall, as development proceeds. Indeed, DTI-based imaging shows that local nodal network *inefficiency* manifests as early as 6 months of age in familially high-risk infants who later are diagnosed with autism, progressing from right primary auditory and middle and superior temporal gyri to higher-order cortices [103].

Perhaps related to this third possibility of discontinuity across the diagnostic boundary is the current result’s place within a mixed bag of previous findings: both functional [28] and structural [30] imaging studies have identified *reduced* average nodal resting-state functional network efficiency as a function of autistic traits in non-autistic adults [28], driven by inefficiencies in the default-mode network centred on posterior cingulum. However, structural imaging in categorically autistic children shows the opposite relationship, heightened efficiency with increasing autism severity [30]. Studies of autistic traits and simple functional connectivity, not deriving network metrics, have found mixed results in which autistic traits are related to lesser functional connectivity between a rostral anterior cingulate region of interest and bilateral mid-insula [29] but greater functional connectivities between the same region of interest and other insular subregions [29, 104] as well as lateral occipital cortex, superior parietal cortex and angular gyrus [29], and perhaps developmentally related to lesser connectivity within the default-mode network centred on posterior cingulum [104]. One study identified opposite occipitofrontal connectivity perturbations in two subgroups [105]. To the extent that this collection of methods and results can support any general conclusion about functional network characteristics and autistic traits, that conclusion seems the negative one that this relationship is not necessarily monotonic across the autism diagnostic boundary and/or across development, and that these dependencies may differ between brain networks. Indeed, recent theoretical work points out that in this context of normal individual differences in cognition, so-called ‘autistic’ trait dimensions, defined so generally as to encompass much individual cognitive variation, may well be influenced by mechanisms distinct from those that produce the syndrome of autism [11].

Our DTI data, in contrast to fMRI, offer by their nature a more granular description of connectivity, highlighting specific anatomical nodes of convergence associated with contextual association (tapped by the Systemizing Quotient) and theory-of-mind in particular. In this analysis, greater network integrity of a functional brain region reflects greater ability within the corresponding domain of cognitive function, regardless of whether such ability maps onto greater or lesser levels of autistic traits.

It seems especially notable that this analysis confirms right supramarginal gyrus in particular as a driver of faster perspective-taking, a result consistent with experimental evidence relating the right temporoparietal junction to allocentric perspective-taking [106] and attribution of mental states [31]; the posterior inferior extent of this AAL-90 region is consistent with the localisation of theory-of-mind activations [107], although an analysis in individual subject space could provide more specific anatomical confirmation. Likewise notable is the absence, within these functional and structural graph measures, of any association of right supramarginal gyrus with the RMET, contrary to the case of simple correlative analysis with task-based fMRI [108].

Interpretation of the SQ’s bilateral medial temporal localisation is complicated by the persistent ambiguity as to what skill or trait it is that the SQ is measuring [39], although we have argued (*vide supra*) that the SQ measures invariant contextual association. Like the slowed attentional orienting discussed above, invariant contextual association can be straightforwardly associated with autistic *bricolage*, the building up of configural representations from their component parts and details. And indeed contextual association in general has been proposed [109, 110] as a parsimoniously unifying theme for parahippocampal cortex’s functional associations with mapping and navigation as well as episodic memory, all skills associated with autism. The computational structure of the medial temporal lobe can support representations of spatial, temporal, and conceptual distances and contexts [111]; autistic differences in synaptic strengths might bias hippocampal firing sequences towards representation of short distances, producing a knack for local detail.

## Limitations

Interpretation of these relationships between brain connectivity and behavioural measures is complicated many meanings of the term ‘connectivity’ [88, 112], referring variously to functional connectivity (correlated time series), anatomical connectivity (tracts and synapses) and computational connectivity (mutual information), on multiple spatial and temporal scales. Indeed, the question of connectivity differences in autistic versus non-autistic brains, or in this case as a function of individual differences in autistic traits, depends at least as much on what we look for and how we look for it as it does on what we are looking at.

Individual functional mapping both of task-related activations within delimited brain areas [113] and of resting-state network correlations across the whole brain [83] shows that individual functional anatomical boundaries are idiosyncratic, and anatomically neighbouring functions are interdigitated when examined on spatial scales finer that gyral and sulcal definitions [83]. Any study that bases and expresses its localisations in terms of central tendencies across individuals, then, can yield only approximate results. Nevertheless, such results do retain an approximate meaning because functional anatomical adjacencies and parallel connections are preserved across individuals, even though their geometric details are not [83]. In terms of the current study, even though a particular AAL-90 parcel will in general include more than a single functional brain region, at a population (and sample) level, this blurring of several individual functional anatomies into each AAL-90 parcel does not impair power to make inferences about the functional anatomical neighbourhood of that parcel (*e.g.,* [114–116]), and to support hypotheses that localise a functional region wholly or probabilistically to a particular parcel, e.g. theory-of-mind whose right temporoparietal hypothesis falls within AAL-90’s right supramarginal gyrus parcel.

No matter how powerful the physiological assays, brain-behaviour correlations can be only as sensitive and specific as the behavioural measures are. Of especial relevance to this current work, quantitative assessment of sensory sensitivity remains a developing endeavour, in which survey reports such as the SSQ may not converge with direct observations [117]. Likewise, the story surrounding FC-EFT and EFT variants in general as a measure of autistic perceptual focus on detail has become more and more equivocal [54], and the RMET seems to accomplish its correlation with autistic traits more by measuring verbal skills than by measuring empathy *per se* [50, 51].

## Conclusions

This study associates more autistotypal (i.e. more impaired) levels of social responsiveness and attention orienting with greater efficiency of brain-wide functional networks, and greater levels of systemising and of social perspective-taking with greater structural network efficiencies centring on medial/anterior temporal lobe and right temporoparietal junction, respectively. The skills indexed by widespread functional network efficiency may be more general across social and non-social domains of cognition, and those indexed by anatomically specific structural network efficiency more domain-specific. This study has not investigated intra-individual variation in the moment-to-moment dynamics of functional brain connectivity [118, 119], an approach that in future may help to resolve apparent inconsistencies between these and other early results on dimensional relationships between autistic traits and neural connectivity. Further work can complement the current results with EEG or MEG imaging modalities sensitive to high-frequency signals and can resolve the open questions of whether these relationships between autistic traits and brain network efficiencies are invariant across development, and across the diagnostic boundary between autistic traits and autism spectrum conditions. Understanding how people with autistic traits think can ultimately help inform individualised supports for people within and beyond the autism spectrum.

## Supplementary Information

**Additional file 1.** Behavioural data. Spreadsheet of behavioural data for all subjects; and log data from each subject’s theory-of-mind test, in raw (ExperimentLog.txt) and tabular (.csv) formats.

**Additional file 2.** Structural images 1-7. Anonymised T1 structural images for subjects 1 to 7, in gzipped NIfTI format.

**Additional file 3.** Structural images 8-14. Anonymised T1 structural images for subjects 8 to 14, in gzipped NIfTI format.

**Additional file 4.** Structural images 15-21. Anonymised T1 structural images for subjects 15 to 21, in gzipped NIfTI format.

**Additional file 5.** Structural images 22-27. Anonymised T1 structural images for subjects 22 to 27, in gzipped NIfTI format.

**Additional file 6.** Structural images 28-30. Anonymised T1 structural images for subjects 28, 29, and 30, in gzipped NIfTI format.

**Additional file 7.** Nuisance regressors. Nuisance regressor time series for each subject, as floating-point values in ASCII text format.

**Additional file 8.** Connectivity matrices. Connection matrices and functional correlation matrices for each subject, as floating-point values in ASCII text format, and MATLAB code for producing these.

**Additional file 9.** Brain-behaviour correlations. README file detailing data curation; AAL-90 region masks in NIfTI format; spreadseehts of Spearman correlations; code and data for the correlational analysis.

**Additional file 10: Supplementary Methods.** Theory-of-Mind Measurement.

## Data Availability

Pseudonymised behavioural data and brain-behaviour correlations will be made available as supplementary files alongside this manuscript. Ethical permission and consent do not allow for sharing individual subjects’ fMRI and DTI brain images.
