# Supplementary material for: Autistic traits and individual brain differences: functional network efficiency reflects attentional and social impairments, structural nodal efficiencies index systemising and theory-of-mind skills
Source: Mol Autism. 2021 Jan 21;12:3. doi: 10.1186/s13229-020-00377-8 (PMC7818759; doi:10.1186/s13229-020-00377-8)
Supplement: Supplementary file 10 — Additional file 10: Supplementary Methods. Theory-of-Mind Measurement. [file 13229_2020_377_MOESM10_ESM.pdf]

Supplementary Methods: Theory-of-Mind Measurement

Matthew K. Belmonte

First- and second-order theory-of-mind (ToM) were assessed via a version of the 'Sally Anne' test embedded as one of several mini-games within our 'Astropolis' suite of computer game measures for quantitative assessment of autistic attentional, executive and social cognitive functioning (Yoder & Belmonte, 2010; www.AutismCollaborative.org). (Other Astropolis mini-games were not played in this study.) The game was designed to depend primarily on iconic video vignettes and only secondarily on textual narrative that supplemented this visual information. In vignette #1, a friendly captain in her spaceship deposits cargo on a randomly selected one of four available planets arranged at the top, bottom, left and right of the display. Planets are distinguishable by their locations within the display and by their colours and textures. Friendly characters in a blue motif are distinguished from the pirate in red. Video stimuli are followed by brief textual summaries in which nouns (characters, spaceships, cargo, planets) are supplemented with the corresponding icons. In vignette #2, a pirate in his spaceship flies into the picture and either steals the cargo and deposits it on another randomly selected planet distinct from the first planet (theft, 2/3 probability) or simply scans the cargo (no theft, 1/3 probability). Vignette #3 pans away from the planets to a flagship, where a friendly admiral is watching a viewscreen. The admiral's viewscreen shows either a sensor scan of the pirate's actions (observed, ½ probability) or noise representing sensor jamming (unobserved, ½ probability). Thus by the end of vignette #3, one of three equiprobable situations exists: (1) the captain believes correctly that the cargo is in its original location, and the pirate believes correctly that the captain will look for the cargo in this location (no theft); (2) the captain believes incorrectly that the cargo is in its original location, and the pirate believes correctly that the captain will look for the cargo in its original location (unobserved theft); (3) the captain believes correctly that the cargo is in its new location, and the pirate believes incorrectly that the captain will look for the cargo in its original location (observed theft).

Instruction pages remind the player that the captain will always set course to retrieve the cargo, and that the pirate will always set course to find the captain. After acknowledging these reminders with keypresses, the player is prompted with the first-person question "Where will you send a ship to meet up with the captain? Use an arrow key to choose a planet" and then the third-person question "Where will you send a ship to intercept the pirate?" Use an arrow key to choose a planet". To choose the correct planet the player must infer where the captain thinks the cargo is and where the pirate thinks the captain will look for the cargo,

respectively. Feedback for each choice is given after both the first-person and the third-person choices within a trial have been completed. Response times in each trial are measured as the interval between the end of vignette #3 and the first-person keypress (thus including the time taken by the reminders), and between the presentation of the third-person question and the third-person keypress. Egocentric first-order theory-of-mind latency is computed as the mean first-person response time in the unobserved theft condition minus that in the no-theft condition (modelling the captain's true versus false belief). Allocentric (Frith & de Vignemont, 2005) first-order theory-of-mind latency is computed as the mean third-person response time in the unobserved theft condition minus that in the no-theft condition (putting oneself in the place of the pirate to model truly the captain's false versus true belief). Second-order theory-of-mind latency is computed as the mean second-order response time in the observed theft condition minus that in the unobserved theft condition (modelling the pirate's false versus true belief about the captain's belief). In all these computations, any trials with incorrect responses are excluded. Some subjects took inordinately long on the very first trial; for subjects for whom the first trial's reaction time was more than two standard deviations away from the mean reaction time of all the other trials in the same condition, the first trial was excluded.

Conditions in each trial are selected randomly according to the aforementioned probabilities. Because this random selection of condition is hardcoded into the software, it was not possible to specify uniform numbers of trials in each condition for all subjects. Trials were run for a total of 30 minutes. After the aforementioned exclusions for incorrect responses and for first-trial outliers, subjects had between 1 and 7 (mean 3.3) no-theft trials, between 1 and 6 (mean 2.3) unobserved theft trials, and between 0 (in six subjects, who were excluded from the second-order analysis) and 5 (mean of non-zero counts 2.2) observed theft trials. Our experience with this task has been that even small numbers of trials can produce informative measures of speed of theory-of-mind inference.
