# Supplementary material for: Autistic traits and individual brain differences: functional network efficiency reflects attentional and social impairments, structural nodal efficiencies index systemising and theory-of-mind skills
Source: Mol Autism. 2021 Jan 21;12:3. doi: 10.1186/s13229-020-00377-8 (PMC7818759; doi:10.1186/s13229-020-00377-8)
Supplement: Supplementary file 9 — Additional file 9. Brain-behaviour correlations. README file detailing data curation; AAL-90 region masks in NIfTI format; spreadseehts of Spearman correlations; code and data for the correlational analysis. [file 13229_2020_377_MOESM9_ESM.zip › Paul_Arora_et_al_data/Physiological/README.doc]

**The following column names have been used for the behavioural variables. These behavioural variables are mentioned as the column names of the results files. The entries on the right hand side of the ‘=’ sign are the columns of the Manesar_data_updated.xls file. Dr. Matthew has suggested those columns to consider for brain-behaviour association study.**

Verbal_IQ=I; Performance_IQ=J;

(Also included IQ_Full)

Item_1A=(AE-R)./R; Item_1B=(AE-R)

Item_2A=(CZ-CG)./BT; Item_2B=(CZ-CG);

Item_3A=(CG-BT)./BT; Item_3B=(CG-BT);

Item_4A=DZ+EA+EB+EC; Item_4B=DT+DU+DV+DW;

Item_5A=ED; Item_5B=DX;

EQ=EF;

SQ=EH;

SSQ_Total=EL;

SRS=ER;

RMET_CORRECT=EW;

COWA_TOTAL_Production=FP;

AnNT_Production=FW;

FC_EFT_QAMean=GH;

ANT_ALERT=HI;

ANT_ORIENTING=HK;

ANT_CONFLICT=HM;

ANT_GRANDMEAN=HO;

**Age, gender and total brain volume were regressed out from the brain network measures using GLM framework. Two tailed spearman correlations involving 10000 permutations has been used to correlate the behavioural measures and residuals of brain network measures. Brain volumes were estimated using SIENA tool of FSL. Two types of brain network measures have been used: global and nodal. Global network measure has one value per subject. Nodal measures have 90 values for 90 cortical regions / subject. I have used three global measures (clustering coefficient, characteristic path length, efficiency) and three nodal measures (efficiency, clustering coefficient and efficiency). The interpretations of these measures are available at** <http://www.sciencedirect.com/science/article/pii/S105381190901074X?via%3Dihub>

**The results/spearman_Correlation folder contains two sub folders: global and local**

**Global folder contains spearman correlation coefficient (r) and uncorrected p values corresponding to the correlation between the DTI/rsfMRI based global network measures and behaviour variables. Significant (p<0.05) results have been highlighted with red colour. Correlation coefficients (r) and p-values have been saved in different files.**

**Nodal folder contains r and p values for different nodal measures in different files. We have performed FDR correction at (p<0.05) threshold. The name of the behaviour column has been colored in red. If you go down the column , you will see uncorrected p values. If you further go down, you will see the serial number of brain regions which are significantly associated with that behaviour at pFDR<0.05 level.**

**The serial number associated with the brain regions are listed below on the left side of the brain region names:**

1 Precentral_L

2 Precentral_R

3 Frontal_Sup_L

4 Frontal_Sup_R

5 Frontal_Sup_Orb_L

6 Frontal_Sup_Orb_R

7 Frontal_Mid_L

8 Frontal_Mid_R

9 Frontal_Mid_Orb_L

10 Frontal_Mid_Orb_R

11 Frontal_Inf_Oper_L

12 Frontal_Inf_Oper_R

13 Frontal_Inf_Tri_L

14 Frontal_Inf_Tri_R

15 Frontal_Inf_Orb_L

16 Frontal_Inf_Orb_R

17 Rolandic_Oper_L

18 Rolandic_Oper_R

19Supp_Motor_Area_L

20Supp_Motor_Area_R

21 Olfactory_L

22 Olfactory_R

23Frontal_Sup_Medial_L

24Frontal_Sup_Medial_R

25Frontal_Med_Orb_L

26Frontal_Med_Orb_R

27Rectus_L

28Rectus_R

29Insula_L

30Insula_R

31Cingulum_Ant_L

32Cingulum_Ant_R

33Cingulum_Mid_L

34Cingulum_Mid_R

35Cingulum_Post_L

36Cingulum_Post_R

37Hippocampus_L

38Hippocampus_R

39ParaHippocampal_L

40ParaHippocampal_R

41Amygdala_L

42Amygdala_R

42Calcarine_L

44Calcarine_R

45Cuneus_L

46Cuneus_R

47Lingual_L

48Lingual_R

49Occipital_Sup_L

50 Occipital_Sup_R

51 Occipital_Mid_L

52Occipital_Mid_R

53Occipital_Inf_L

54Occipital_Inf_R

55Fusiform_L

56Fusiform_R

57Postcentral_L

58Postcentral_R

59Parietal_Sup_L

60Parietal_Sup_R

61Parietal_Inf_L

62Parietal_Inf_R

63SupraMarginal_L

64SupraMarginal_R

65Angular_L

66Angular_R

67Precuneus_L

68Precuneus_R

69Paracentral_Lobule_L

70Paracentral_Lobule_R

71Caudate_L

72Caudate_R

73Putamen_L

74Putamen_R

75Pallidum_L

76Pallidum_R

77Thalamus_L

78Thalamus_R

79Heschl_L

80Heschl_R

81Temporal_Sup_L

82Temporal_Sup_R

83Temporal_Pole_Sup_L

84Temporal_Pole_Sup_R

85Temporal_Mid_L

86Temporal_Mid_R

87Temporal_Pole_Mid_L

88Temporal_Pole_Mid_R

89Temporal_Inf_L

90Temporal_Inf_R
